# Insights into amino acid fractionation and incorporation by compound-specific carbon isotope analysis of three-spined sticklebacks

**DOI:** 10.1038/s41598-022-15704-7

**Published:** 2022-07-08

**Authors:** Tobias Hesse, Milen Nachev, Shaista Khaliq, Maik A. Jochmann, Frederik Franke, Jörn P. Scharsack, Joachim Kurtz, Bernd Sures, Torsten C. Schmidt

**Affiliations:** 1 Instrumental Analytical Chemistry, University of Duisburg-Essen, Universitätsstr. 5, 45141 Essen, Germany; 2 Aquatic Ecology, University of Duisburg-Essen, Universitätsstr. 5, 45141 Essen, Germany; 3 Centre for Water and Environmental Research, University of Duisburg-Essen, Universitätsstr. 5, 45141 Essen, Germany; 4 Institute for Evolution & Biodiversity, University of Münster, Hüfferstr. 1, 48149 Münster, Germany; 5 Present Address: Thünen Institute of Fisheries Ecology, Herwigstr. 31, 27572 Bremerhaven, Germany; 6 Present Address: Bavarian State Institute of Forestry, Hans-Carl-von-Carlowitz-Platz 1, 85354 Freising, Germany

**Keywords:** Stable isotope analysis, Ecology

## Abstract

Interpretation of stable isotope data is of upmost importance in ecology to build sound models for the study of animal diets, migration patterns and physiology. However, our understanding of stable isotope fractionation and incorporation into consumer tissues is still limited. We therefore measured the δ^13^C values of individual amino acids over time from muscle and liver tissue of three-spined sticklebacks (*Gasterosteus aculeatus*) on a high protein diet. The δ^13^C values of amino acids in the liver quickly responded to small shifts of under ± 2.0‰ in dietary stable isotope compositions on 30-day intervals. We found on average no trophic fractionation in pooled essential (muscle, liver) and non-essential (muscle) amino acids. Negative Δδ^13^C values of − 0.7 ± 1.3‰ were observed for pooled non-essential (liver) amino acids and might indicate biosynthesis from small amounts of dietary lipids. Trophic fractionation of individual amino acids is reported and discussed, including unusual Δδ^13^C values of over + 4.9 ± 1.4‰ for histidine. Arginine and lysine showed the lowest trophic fractionation on individual sampling days and might be useful proxies for dietary sources on short time scales. We suggest further investigations using isotopically enriched materials to facilitate the correct interpretation of ecological field data.

## Introduction

Stable isotope analysis (SIA) of carbon is a powerful tool in ecological studies to investigate resource utilization, foraging behavior and migration patterns of animals^1–3^. The underlying principle is that the carbon stable isotope composition (δ^13^C) of diets is mostly retained by consumers, with only small amounts of trophic fractionation occurring during incorporation and metabolism of nutrients. In bulk stable isotope analysis (BSIA), trophic fractionation therefore only increases the δ^13^C values of consumers by 0–1‰^4, 5^ and the carbon stable isotope signature of an individual is mostly depending on the isotope composition of primary producers. This enables tracking of nutrients from the base of food webs to higher trophic level predators. However, there can be deviations from this pattern depending on species, analyzed tissue and diet composition^6, 7^. A more recent approach, compound specific stable isotope analysis (CSIA), was enabled by analyzing individual compounds rather than bulk tissues. Where BSIA of carbon typically shows little fractionation between diet and consumer, individual constituents such as amino acids (AA) can have higher fractionation, depending on which compounds they are routed from and the nutrient composition of diets^3, 8–12^.

AAs can be divided into essential amino acids (EAA) and non-essential amino acids (NEAA, Table 1). EAAs cannot be synthesized by higher organisms and therefore need to be taken up directly from dietary sources, leading to no or very little trophic fractionation as they are traversing the food chain mostly unchanged from primary producers to top predators^3, 13–15^. NEAAs on the other hand can be synthesized de novo in higher organisms and therefore originate either from dietary protein and/or other macronutrients, such as lipids and carbohydrates. Whether these compounds are directly routed or synthesized de novo is depending on dietary nutrient composition, e.g. consumers fed with high protein contents tend to also directly incorporate NEAAs in order to preserve energy^11, 13, 16, 17^, whereas biosynthesis of NEAAs from lipids leads to lower δ^13^C values because lipids are depleted in ^13^C compared to proteins or carbohhydrates^18, 19^. However, the classification of AAs into essential and non-essential is not always clear, as nutrient requirements of some NEAAs, e.g. glutamic acid (Glx) and proline (Pro), can outmatch an organism’s ability to synthesize these compounds, rendering them temporarily or conditionally essential^20^. Another special case would be tyrosine (Tyr), which can be synthesized de novo in higher organisms but is directly derived from phenylalanine (Phe), which is an EAA. The carbon isotope signature of Tyr therefore typically falls into the same range as Phe^12^, although it should be considered non-essential.

**Table 1:** Classification of analyzed AAs in fish into essential/non-essential and glucogenic/ketogenic. Abbreviations are given in brackets. Adapted from Falco et al. (2020)^72^.

	Glucogenic	Glucogenic/ketogenic	Ketogenic
Essential	Arginine (Arg)	Phenylalanine (Phe)	Lysine (Lys)
	Histidine (His)		
	Threonine (Thr)		
Nonessential	Alanine (Ala)	Tyrosine (Tyr)	
	Asparagine/aspartate (Asx)		
	Glutamine/glutamate (Glx)		
	Glycine (Gly)		
	Proline (Pro)		
	Serine (Ser)		

One shortcoming of most ecological studies using stable isotopes is that they typically only consider whole body or muscle tissue^21^, as these are easy to access. The turnover and incorporation rate in muscle tissue is rather low and more affected by consumer physiology and growth phases during which protein synthesis and deposition occurs^22, 23^. The stable isotope signature of muscle tissue therefore reflects the long-term dietary intake and remains conservative towards small or only seasonal changes in dietary δ^13^C values. The liver, on the other hand, responds more quickly to small or seasonal dietary changes, as its regulatory activities require continuous protein turnover^22–26^. In addition, the liver plays an important role in the metabolism and biosynthesis of AAs^20, 27, 28^, which makes it an ideal tissue for studying these processes. Another shortcoming of most studies using stable isotopes is that they are field based, hence there was an initial call for more laboratory studies in 1997^29^ to understand the fundamental principles of isotopic incorporation, trophic discrimination and isotope routing. Although the number of laboratory-based studies has increased since then, the call was renewed in 2009^30^ to facilitate correct interpretation of field data. Few controlled feeding experiments so far examined the carbon isotope fractionation of individual AAs between diet and fish consumers, with varying magnitudes and directions of trophic fractionation reported^10–13, 17^. McMahon et al. (2010) found ^13^C-depleted isotope signatures of Gly between Common Mummichogs (*Fundulus heteroclitus*) and one of their diets, whereas Rogers et al. (2019) found significantly ^13^C-enriched Gly stable isotope signatures of Chinook Salmon (*Oncorhynchus tshawytscha*) reared on the same diet. This example demonstrates that clear differences in individual AA trophic fractionations can occur among fish species.

The three-spined stickleback (*Gasterosteus aculeatus*) is a well-studied model fish in ecology, evolutionary biology and parasitology^31, 32^, yet no carbon stable isotope analysis of AAs has been done to this date. The only studies reported so far are field-based and use BSIA to investigate sex, armor, phenotypes, genetics and host-parasite relationships^33–36^. We conducted a laboratory-based feeding experiment, where three-spined sticklebacks were reared on a protein rich diet with low amounts of lipids (60% protein, 5% lipids) over the course of four months. To contribute to our understanding of isotope incorporation, discrimination and routing, we measured the carbon stable isotope signature of individual AAs from muscle, liver and dietary samples taken on 30-day intervals by Liquid Chromatography Isotope Ratio Mass Spectrometry (LC-IRMS). Previous BSIA of dietary samples (data not published) revealed a minor carbon stable isotope shift from − 15.8‰ after 30 days to − 17.0‰ after 60 days, − 18.1‰ after 90 days and − 16.9‰ after 120 days, which is likely transferred to fish tissue to varying degrees depending on tissue type. Based on our current knowledge of isotope incorporation, fractionation and the high protein contents in fish diets, we expect that both EAAs and NEAAs in muscle and liver tissue of sticklebacks will be mainly routed from dietary sources and therefore show little trophic fractionation. Furthermore, if the dietary stable isotope shift over time from BSIA is also measurable using CSIA of individual AAs, we expect that the high protein turnover of liver tissue will also result in a significant isotope shift between sampling days in contrast to muscle tissue. Lastly, the small amounts of other macronutrients than dietary protein might not be enough to result in significantly different δ^13^C values between liver and dietary samples, but using multivariate analysis might indicate patterns of fractionation due to biosynthesis or metabolism of NEAAs. This is the first time that carbon isotope signatures of AAs were measured for three-spined sticklebacks on a time series and in a controlled feeding experiment. Our goals were to investigate δ^13^C values of AAs from liver and muscle tissue, especially in response to changing dietary δ^13^C values over time and to find differences in δ^13^C patterns between tissues. Furthermore, we expect that NEAAs and EAAs will show similar and small trophic fractionation in Δδ^13^C values in general, since fish are fed on high protein contents. Our results will therefore help to validate common assumptions in CSIA-AA, but also show limitations and new possibilities in ecology to interpret the carbon stable isotope signatures of AAs from different tissue types and time intervals.

## Results

### AA δ^13^C changes between sampling days

One-way analysis of variance (ANOVA) for each AA with sampling days as independent variable and δ^13^C values as dependent variable of dietary samples revealed a significant isotope shift over time for all AAs except His (DF = 3, 8; *p* < 0.01; Table S2). A trend can be seen where the carbon stable isotope signature of each dietary AA decreased between 30 and 60 days as well as 60 and 90 days, followed by an increase in δ^13^C values between 90 and 120 days (Fig. S2), although the shift was not always significant between consecutive sampling days (30–60, 60–90, 90–120 days). The highest differences between − 2.4 and − 4.2‰ were observed between 30 and 90 days. Isotope signatures of AAs in the liver were significantly different over time for Ala (F_3,8_ = 6.8, *p* = 0.004), Asx (F_3,8_ = 9.2, *p* = 0.001), Arg (F_3,8_ = 13.7, *p* < 0.001), Lys (F_3,8_ = 18.4, *p* < 0.001), Phe (F_3,8_ = 8.9, *p* = 0.001) and Tyr (F_3,8_ = 20.8, *p* < 0.001). Most of these differences were also observed between 30 and 90 days, with the addition of Glx between those specific sampling days and decreasing δ^13^C values between − 2.1 and − 3.0‰. Although not all AAs in the liver revealed a significant isotope shift, the trend of decreasing δ^13^C values from 30 to 60 days and from 60 to 90 days followed by an increase from 90 to 120 days was still comparable to dietary samples. No significant differences between sampling days were observed for muscle tissue for any AA.

### Trophic fractionation of NEAAs, EAAs and individual AAs among liver and muscle

We investigated trophic fractionation between NEAAs and EAAs over the complete sampling period. Two-way ANOVA on Δδ^13^C values of pooled NEAAs and EAAs (NEAA/EAAs and tissue as fixed factors, Table S4) revealed no significant difference in trophic fractionation between NEAAs and EAAs (F_1, 435_ = 5.6, *p* = 0.019, α = 0.01) among all samples, but there was a significant interaction between factors (F_1, 435_ = 9.4, *p* = 0.002). The interaction was caused by negative Δδ^13^C values of − 0.7 ± 1.3‰ for NEAAs in liver tissue compared to Δδ^13^C values of − 0.1 ± 1.1‰, 0.0 ± 1.4‰ and 0.0 ± 1.1‰ for EAAs in liver and both NEAAs and EAAs in muscle, respectively, resulting in significant differences in trophic fractionation around ± 0.8‰ between those samples (Tukey’s tests, Table S4). Differences in trophic fractionation between EAAs in the liver and NEAAs and EAAs in muscle were not observed.

**Figure 1:**
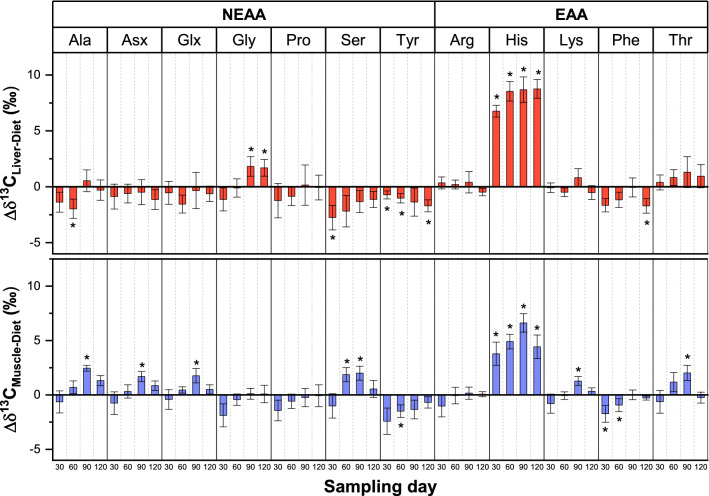
Carbon stable isotope signatures of AAs show low trophic fractionation between fish liver/muscle and dietary samples except for His. Trophic fractionation between stickleback and dietary samples was estimated by calculating Δδ^13^C values ± SD (error bars, n = 5) for liver () and muscle ( ) samples. Asterisks over Δδ^13^C values indicate significant differences from two-sided t-tests against 0 (*p* < 0.01, Table S3). Δδ^13^C values are generally below ± 2‰ for all AAs in muscle and liver samples except for His. The frequently significant Δδ^13^C values in muscle samples on day 90 are caused by the low protein turnover and therefore minor decrease of δ^13^C values in muscle samples compared to the significant δ^13^C decrease in dietary samples. Arg and Lys have the lowest trophic fractionation overall.

Δδ^13^C values of individual AAs between fish tissues and diets were calculated over the complete sampling period and on each sampling day (Fig. 1, Table S3). Significant differences were tested with two-sided t-tests for each value (N = 20 for the complete sampling period, N = 5 on each sampling day) and with a confidence level of 0.01. Significant negative Δδ^13^C values over the complete sampling period were measured for Asx (− 0.8 ± 1.0‰), Glx (− 0.8 ± 1.1‰), Ser (- 1.8 ± 1.2‰), Tyr (− 1.2 ± 0.8‰) and Phe (− 1.1 ± 0.9‰) in liver tissue and for Tyr (− 1.5 ± 1.0‰) and Phe (− 0.7 ± 0.9‰) in muscle tissue, whereas positive Δδ^13^C values were measured for Ala in muscle (1.0 ± 1.3‰), Thr in liver (0.9 ± 1.0‰) and His in both tissues (8.2 ± 1.2‰ and 4.9 ± 1.4‰, respectively). In some of the mentioned cases, average Δδ^13^C values are lower than the measured SD, but due to the larger sampling size (N = 20 over the complete sampling period) the resulting p-values were still below the significance level of 0.01.

The highest Δδ^13^C values on individual sampling days in liver tissue were measured for His (6.8 ± 0.5‰ on day 30, 8.5 ± 0.9‰ on day 60, 8.7 ± 1.1‰ on day 90 and 8.7 ± 0.8‰ on day 120), for Gly on day 90 (1.8 ± 0.9‰) and day 120 (1.7 ± 0.7‰) and for both Tyr (− 0.7 ± 0.4‰ on day 30, − 1.0 ± 0.4‰ on day 60 and − 1.7 ± 0.5‰ on day 120) and Phe (− 1.7 ± 0.6‰ on both day 30 and 120). Significant Δδ^13^C values were measured in muscle tissue on day 90 for Ala (2.5 ± 0.3‰), Asx (1.7 ± 0.5‰), Glx (1.8 ± 0.7‰), Ser (2.0 ± 0.6‰), His (6.6 ± 0.9‰), Lys (1.3 ± 0.4‰) and Thr (2.0 ± 0.7‰). Pro, Arg and Lys had the smallest average Δδ^13^C values in both muscle and liver tissue around ± 0.1 to 0.2‰ (Arg, Lys) and around − 0.5‰ (Pro). Except for Lys in muscle on day 90, no significant fractionation on individual sampling days were measured for those three AAs. The most significant isotope fractionation was observed for His between diet and both liver and muscle. Since peak areas of His were low compared to the earlier and closely eluding Lys (Fig. S1), we tested different background algorithms (individual, dynamic and manual) in the Isodat 2.0 software to check if the differences between His δ^13^C values could be explained by interferences and coelution of the more abundant Lys. Regardless of the used peak integration and background detection procedure, a strong isotope fractionation of His between dietary and liver/muscle was always observed.

### Patterns of AA δ^13^C values among tissues

**Figure 2:**
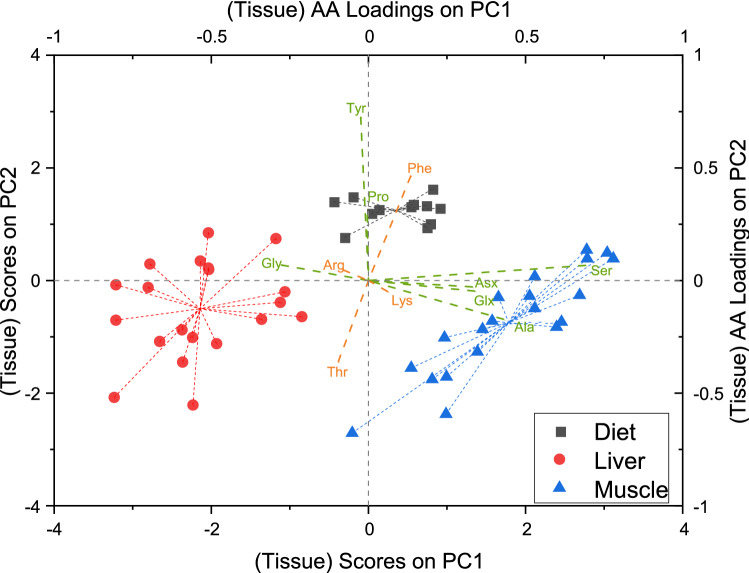
Multivariate analysis of δ^13^C values shows distinct δ^13^C patterns of NEAAs in liver samples compared to dietary and muscle samples. Biplot of sample scores from ASCA for factor 1 (tissue) separates between liver () and muscle () or dietary () samples on PC1. Scores of liver samples on PC1 are negative in contrast to positive scores of muscle samples or scores around 0 for dietary samples. NEAA loadings () on PC1 are positive for Ala, Ser, Asx and Glx and negative for Gly, whereas EAAs () and Pro have no impact on PC1. AA loadings on PC2 are positive for Pro, Phe and Tyr and negative for Thr.

Since differences in δ^13^C values between fish and dietary samples might be low in response to the used high protein diets, we performed ANOVA simultaneous component analysis (ASCA) to investigate patterns of AA δ^13^C values in a multivariate approach. Sample scores of liver tissue were separated from muscle and dietary samples on the first principal component of factor 1 (Tissue, Fig. 2), which contributed 22.4% to the total variance. NEAAs were the main driver of separation with loadings above 0.3 for Ala, Asx, Glx and Ser, whereas Gly was the only NEAA with a negative loading of below − 0.2 (Fig. 2). Ser had the overall highest loading of 0.7 on PC1. All EAAs and Pro had loadings of below ± 0.2 and therefore little to no effect on PC1 separation. Individual scorings of liver samples were negative on PC1, whereas muscle and dietary samples had almost exclusively positive scorings. PC2 showed slight separation between fish tissue and diets and was mostly loaded with the EAAs Phe, Thr as well as the NEAAs Pro and Tyr (Fig. 2). Arg and Lys were the only AAs which had no impact on separation between tissues for both PCs. Factor 2 (sampling days) of the ASCA had an effect of 35.5% on system variance and PC1 accounted for 93.5% of variance on factor 2. AA loadings on PC1 were exclusively positive between 0.15 and 0.4. Sample scores centered around + 4 for samples taken after 30 days, 0 for samples after 60 days, − 3 for samples after 90 days and − 1 for samples after 120 days, but clear separation of samples was not achieved in a score plot.

## Discussion

The cause for the stable isotope shift of dietary AAs over time in our study remains unknown, but the observed trend of all AAs having a similar shift over time indicates that different batch materials of mosquito larvae might have been used throughout the experiment. The change of δ^13^C values in muscle tissue between any given sampling day was within − 1.8 and + 1.2‰, but these shifts were statistically not significant and overshadowed by natural variations between individuals. This supports the idea that AAs from muscle tissue are a more conservative indicator of long-term dietary intake in fish, similar to what was previously described for BSIA^22, 23^. Liver tissue, on the other hand, showed significant isotopic differences over time, indicating that the high nutrient turnover rate of the liver is reflected in its AA carbon stable isotope signature and even short-term changes are visible. The range of δ^13^C values in the liver was between − 3.3 and + 1.4‰ and EAAs showed the most significant differences (one-way ANOVA, *p* ≤ 0.001), which indicates high turnover for these compounds because they must be rooted from dietary sources. Only Ala and Asx showed significant differences over time from NEAAs in liver tissue. Both AAs are important energy substrates in fish and precursors for gluconeogenesis^37^, which is the metabolic pathway in the liver to synthesize glucose from other carbon substrates. Fish therefore have a high Ala and Asx demand in liver tissue, which might outmatch the fish’s ability to sufficiently synthesize these compounds and lead to an increased incorporation or turnover from dietary sources. According to our results, liver carbon isotope signatures of Ala, Asx, Arg and Lys might be useful proxies to track consumer diets of small teleost fish on protein rich diets and on time scales of at least 30-day intervals. This information could possibly be expanded to non-protein rich diets for Arg and Lys, due to the necessity of eukaryotes to directly incorporate these nutrients from dietary source, regardless of their composition^13^. The change in liver δ^13^C values were most notable between samples from day 30 and 90 and not consistently between consecutive sampling days, which can be attributed to the rather small changes in dietary δ^13^C values of only ± 1 to 2‰ between sampling days, which are also partly overshadowed by natural variations and measurement uncertainties. The isotopic half-life of muscle and organ tissue from ectotherms can be estimated according to their body mass^38^. The average body mass of sticklebacks on sampling days was 1.28 ± 0.35 g (N = 20), which would translate to a half-life of approximately 27 days for muscle and 12 days for liver tissue. This estimation supports the observed significant change of δ^13^C values over time in liver samples and the only slightly decreasing δ^13^C in muscle tissue. The differences in dietary AA δ^13^C values were not consistent between sampling days, which further limits our ability to compare the different trends observed in muscle and liver tissue. However, the most consistent isotope shift was observed during the first 90 days of the experiment with two consecutive negative changes in dietary AA δ^13^C values. These changes are also reflected during that period in liver tissue, which highlights the potential of CSIA to differentiate between even small differences of dietary intake. Although we did not quantify the exact cellular turnover rate of individual AAs in stickleback tissues, knowing an estimated timeline for evaluating the diet of an animal is useful for ecological studies investigating migration or seasonal changes of food sources. It has been shown that choosing different tissue types in individuals enables researchers to explore temporal and spatial resource use of animals and it is critical to consider incorporation rates of isotope signatures into these tissues^39, 40^. Most studies so far on isotopic incorporation and turnover rates are still based on BSIA^41–43^ and enhancing our knowledge to specific compounds in different tissues can provide powerful opportunities to study the physiology of migrating animals, which are hard to capture multiple times. In addition, the analysis of AA isotope signatures of these animals from tissues with different incorporation rates can help to illuminate temporal variations and identify endogenous vs. exogenous resources, without the need of multiple sampling points.

The initial amino acid isotope signature of mosquito larvae used for feeding shows typical patterns also seen in other studies, where Gly and Ser are the most ^13^C-enriched AAs and EAAs are isotopically ^13^C-depleted in comparison to NEAAs^3, 8–13, 15, 17, 44, 45^. NEAAs can be synthesized de novo in higher organisms, leading to isotopic fractionation during metabolism and nutrient flow from primary producers to top predators in a given food web^17^. It is known that the carbon stable isotope composition of NEAA in higher organisms is therefore flexible and varies according to dietary protein and lipid intake. A study from McMahon et al. (2010)^13^ fed common mummichogs on four isotopically distinct diets with different protein and lipid compositions and measured the carbon stable isotope signature of individual AAs from muscle tissue. In two high protein diets based on clam and squid, they found Δδ^13^C values between − 4 and − 7‰ for Ala, Ser and Gly, whereas trophic fractionation was lower for other NEAAs and absent for EAAs. The protein and lipid contents in both diets were 70% and 18%, respectively. Another study from Newsome et al. (2014)^46^ fed rodents diets of different protein and lipid contents with distinct isotope signatures and found that NEAA δ^13^C values in muscle tissue shifted more towards dietary lipids with lower protein/lipid ratios, whereas a protein/lipid ratio of 40%/5% resulted in NEAA δ^13^C close to the protein source. Our study used commercially available dried mosquito larvae with a protein/lipid content of 60% and 5%, respectively, and therefore represents a diet with a higher protein to lipid ratio than previous studies. Our results show low isotope fractionations for NEAAs and EAAs between diets and fish tissue and support the idea of isotope routing when animals are fed protein rich diets, which is energetically favorable compared to de novo synthesis. High isotope fractionations of ± 5‰ of some NEAAs between diet and consumer, as described by other studies^11, 13^, did not occur in our case, probably due to the low abundance of other macronutrients. Fractionation between muscle and dietary samples was visible for Ala, Asx, Glx, Ser, His, Lys and Thr especially on day 90, but these differences are a direct result of muscle tissue not responding to the shifting stable isotope signatures in diets and not due to trophic fractionation. The highest difference in dietary δ^13^C values over time were observed between day 30 and 90 of the feeding experiment. Since muscle tissue has a low turnover rate and is a more conservative indicator of long-term dietary intake, the lack of response to the short-term isotopic shifts in dietary samples results in higher differences between muscle and dietary samples on day 90. Small but significant Δδ^13^C values were observed between liver and dietary samples for Asx, Glx, Ser and Tyr over the whole sampling period and occasionally for Ala and Gly on individual sampling days. The trophic fractionation of Ser in the liver was striking and consistently between − 1 and − 2‰ and Gly was the only NEAA who showed an opposite trend of positive Δδ^13^C values on day 90 and 120. Catabolic pathways for Ser involve deamination to pyruvate, transamination with pyruvate to form hydroxy pyruvate and Ala and formation of Gly with tetrahydrofolate^47^. Additionally, participation of Gly into gluconeogenesis requires the conversion of Gly to Ser by serine hydroxy methyltransferase and both AAs participate further in sulfur and one-carbon metabolism^20^. The liver plays a major role in energy and AA metabolism by regulating and controlling gluconeogenesis as well as synthesizing many of the NEAAs^28^. Synthesis of NEAA carbon backbones can occur from either glycolytic intermediates for Gly, Ser and Ala or from Krebs cycle intermediates for Asx and Glx^48^. It is, however, hard to estimate the contribution of isotopic fractionation for each of these pathways, not only because intrinsic fractionation and mass flow are unknown, but also because metabolic pathways are often intertwined and hard to separate in living organisms at natural abundance level. Enzymatic reactions and catabolism of nutrients are usually associated with discrimination against the heavier isotopes, in this case ^13^C, leading to depleted signatures in the product, while the educt of the reaction becomes isotopically ^13^C-enriched^49^. The small enrichment of Gly observed in liver could therefore be indicative of extensive conversion of Gly to other compounds, with Ser being one possibility and resulting in more noticeable negative Δδ^13^C values. It is, however, a far stretch at this point to account the small isotopic differences in Ser and Gly to specific metabolic or enzymatic reactions, especially since metabolism of Gly and Ser involve several different pathways. Future research and controlled feeding experiments could focus on using isotopically labeled substitutes to track individual metabolic pathways, such as the conversion of Gly and Ser. Pro was the only NEAA in liver tissue showing no trophic fractionation at all over the sampling period, which points more to a behavior like an EAA being routed by diets. As mentioned in the introduction, Pro can be synthesized in higher organisms, but the classification as a NEAA is sometimes misleading as metabolic requirements might heavily outmatch de novo synthesis. Our results indicate that fish fed on high protein contents incorporate Pro mostly from dietary sources leading to no or very little fractionation in both liver and muscle tissue. Arg and Lys were EAAs with very low fractionation overall, strongly following the trend of dietary isotope signatures even in muscle tissue over short time periods. Arg is highly abundant in fish protein and tissue fluid^20^, serving as a precursor for the synthesis of proteins, nitric oxide, urea, Pro, Glx and creatine^50^. Arg is of special interest here because it is not often reported in the literature, since most studies apply GC-IRMS for measurement of carbon stable isotope signatures of AAs, which requires derivatization and results in the loss of Arg^51, 52^. Because of the high abundance, demand and turnover of dietary Arg in fish leading to very little fractionation even in muscle tissue over a short-term dietary isotope shift, it could serve as another proxy in addition to Phe, Leu, Ile and Lys^10–13^ to track carbon sources from primary producers in muscle or liver tissue. Identifying EAAs with very low fractionation patterns is pivotal for ecological studies using isotope fingerprinting to better constrain the source of end-member signatures and study the carbon flow in terrestrial and oceanic environments^3, 8, 9, 13–15, 17^. Phe and His were the only EAAs showing consistently different isotope signatures between diet and fish tissue, which is surprising since they need to be directly taken up and rooted from dietary sources. Phe can be converted to Tyr and the depleted isotopic signature of Tyr compared to diets could therefore also be explained by enzymatic fractionation discriminating against the heavy carbon isotopes. Although EAAs cannot be synthesized by eukaryotes, the fundamental assumption that these constituents must be solely sourced from dietary protein resulting in low isotope fractionation of ~ 0‰ has not always been met. There are studies suggesting that gut microbes can contribute to the homeostasis of EAAs in animals by de novo synthesis, which of course complicates their use as stable and robust proxies for isotope fingerprinting^17, 53–56^. The contribution of microorganisms in the gut of sticklebacks to the EAAs homeostasis could explain the observed difference of Phe and Tyr isotope signatures in fish muscle and liver tissue compared to diets and highlights the importance to experimentally explore the variation of EAA isotope fractionation by the gut microbiome. According to our results, Arg and Lys might therefore be better suited in sticklebacks and possibly other teleost species to estimate their carbon flow and allocate resource consumption, if the contribution of gut symbionts is of no interest. His showed overall the highest fractionation among the analyzed AA. We investigated possible influences of the closely eluding and much more abundant Lys on the δ^13^C values of the less abundant His by varying peak integration and background detection methods in the ISODAT 2.0 software, but found no indication that the high fractionation patterns were caused by chromatographic interferences. Differences in the peak area ratios of Lys/His were different between tissues, but the observed trend of increasing ratios of Lys/His from dietary to liver and finally muscle tissue does not match the increasing δ^13^C patterns from dietary to muscle and then liver tissue. Although we cannot exclude any influence of poor peak resolution between Lys and His and the different ratios on their carbon isotope signatures, we think that these influences would not result in the high fractionation patterns that we observed. This is also supported by an earlier study showing that poor peak resolution in LC-IRMS does not greatly influence the measured carbon isotope signatures, as compared to GC-IRMS^57^. The previously mentioned de novo synthesis and contribution of EAAs from gut microbes could of course be one explanation for the highly ^13^C-enriched isotope signatures of His in in stickleback liver and muscle tissue. Other reasons could include the enzyme histidine decarboxylase, which is produced by bacteria and causes histamine fish poisoning^58, 59^. It has been speculated before that histidine decarboxylase might still be released from autolyzing of bacterial cells during and after freeze drying and convert histidine to histamine^60^. Enzymatic reactions are known to cause isotopic fractionation, which usually discriminates against the heavier isotope^49^ and could result in enriched isotope signature of the remaining histidine in fish tissue. Interestingly, an early study investigating histamine concentrations during storage of flesh and liver tissue of mackerel under different conditions showed a higher increase of histamine concentrations in the liver^61^, which would fit to the higher isotope values of histidine in stickleback liver compared to muscle tissue, if it was caused by enzymatic reactions. Testing this, however, would require histamine isotope measurements in dietary and fish samples to compare isotope signatures and was out of scope of this study. Another explanation would be that sticklebacks fed on mosquito larvae lack dietary histidine to match their metabolic requirements. We did not directly measure the protein and AA content of dietary mosquito larvae, but His has been mentioned in the literature as a more frequent limiting AA when animals are fed with insect meals^62^. His is an important amino acid for growth, tissue formation and hemoglobin synthesis in fish^63^ and a lack of dietary His may lead to increased fractionation in consumer tissue due to catabolism during starvation. There is however no consensus so far on the isotopic effect of starvation or nutritional stress on consumer tissue^64^ especially for single compounds, and such effects could be subject for further studies.

ASCA provided an alternative way to PCA and LDA as classical multivariate analysis and was especially useful in our case because we could incorporate the structure of our experiment into the model, with sampling days and tissue types as two separate factors^65^. This removed a lot of “noise” from the dietary isotope shift when investigating AA δ^13^C patterns between tissue types. All NEAAs except Pro had high positive (Ala, Asx, Glx and Ser) or negative (Gly) loadings on PC1, whereas loadings of EAAs and Pro were low. Sample scores on PC1 were negative for liver samples and either 0 or positive for muscle and dietary samples, which shows that NEAAs in the liver have distinct δ^13^C patterns. Since liver samples had exclusively negative scorings on PC1, NEAAs with positive loadings (Ala, Asx, Glx and Ser) therefore showed a pattern of ^13^C-depletion compared to muscle and diets. As mentioned earlier, the liver plays a major role in biosynthesis of NEAAs, which is dependent on dietary content of macronutrients. Because we used a diet of high protein and relatively low lipid composition, synthesis of NEAA from other nutrients in the liver might be limited but is still visible when combining δ^13^C values of individual AAs in a multivariate approach. Lipids in natural samples typically have lower δ^13^C values compared to AAs or carbohydrates^18, 19^ and partial biosynthesis of NEAAs from lipids in the liver would therefore result in slightly lower δ^13^C values. The observed low loadings of EAAs on PC1 support this assumption since they cannot be synthesized de novo from other macronutrients in the liver and therefore do not show distinct δ^13^C patterns between muscle and liver tissue. Sample scores of factor 2 (sampling days) from ASCA reflected the changing δ^13^C values in samples induced by the dietary isotope shift, with decreasing δ^13^C values during the first 90 days and a small increase after 120 days. Since all AA loadings on factor 2 were positive and within 0.15 to 0.4, the shift of δ^13^C values over time was similar between individual AAs.

## Conclusion

Studying the isotope signature of AAs between diet and consumer gained much attraction during the last years, yet our knowledge of the fundamental principles behind isotope incorporation and fractionation of individual substitutes is still limited. Our study shows that direct isotope routing even of NEAAs might still be the preferred way for nutrient assimilation when fish are fed with high protein diets, and there are not only differences in isotopic turnover rates between muscle and liver, but also different isotopic behaviors that individual AAs in these tissues show based on their metabolic role. However, investigating isotope fractionation of AAs on natural abundance levels might not be the best approach to study fundamental incorporation and turnover patterns, since a lot of information might be lost to natural variations and measurement uncertainty. Although the use of multivariate analysis can help to find general patterns of δ^13^C values between tissue types, we recommend the use of ^13^C-enriched materials in controlled feeding experiments to accurately track metabolic pathways. One good example to investigate could be the mentioned conversion of Gly and Ser or biosynthesis of NEAAs in the liver, which has the potential to greatly improve our understanding of nutrient assimilation and conversion and is long overdue for the correct analysis and interpretation of field data. On the other hand, it is promising to see that even small differences and fluctuations of isotope values, which are more realistically encountered in nature, can be investigated using CSIA of different sample materials.

## Materials and methods

### Feeding experiment

Three-spined sticklebacks were laboratory-raised offspring and reared in twelve 14 L tanks (VewaTech, Germany) as part of a parasitic infection experiment. Water was recirculated and held at 18 °C with a 15 h light and 9 h dark cycle. Stickleback offspring were produced by in vitro fertilization from individuals collected from a brook in North-West Germany (52° 17′ 33.11″ N, 7°36′ 46.48″ E), about eight months old at the beginning of the experiment and fed daily with washed red mosquito larvae (Chironomidae) over the course of four months. Each lab tank contained twelve individuals, which were divided into two groups. The first group consisted of three individuals per tank, which were exposed to an uninfected copepod as a sham-exposed control group, while the other nine individuals per tank were exposed to an infected copepod as a parasite-exposed group. Three complete lab tanks with 36 individuals were sampled on each sampling date (30, 60, 90 and 120 days post exposure) and five out of the fifteen sham-exposed individuals per sampling date were randomly selected for CSIA. Sub samples of mosquito larvae were taken once a week, stored at − 20 °C and pooled between 1–30, 31–60, 61–90 and 91–120 days for CSIA. Dried red mosquito larvae are commercially available and a protein rich diet for fish, with a crude protein content of up to 60% and a crude lipid content of around 5%. The AA profile is balanced and adequate for fish farming, although His, Lys or Try can be lacking depending on the used insect species^62, 66, 67^. Sticklebacks were starved 72 h prior sampling, anesthetized with MS 222 (Sigma-Aldrich, USA) and killed by decapitation. Liver and muscle tissue were collected without skin and bones and stored at − 20 °C until further use. Sticklebacks were maintained and treated in accordance with the local animal welfare authorities and the EU Directive 2010/63/EU for animal experiments. All animal experiments described were approved by the ‘State Agency for Nature, Environment and Consumer Protection’ (LANUV) of North Rhine Westphalia, which includes the evaluation by an ethics committee, under the project number 87 51.04.2010.A297. The present study was carried out in compliance with the ARRIVE guidelines (https://arriveguidelines.org/).

### Sample preparation

Hydrolysis of amino acids for LC-IRMS analysis has been described in the literature ^44, 45, 68^. Approximately 5 mg of sample material were weighed into 5 mL PTFE vials (CEM GmbH, Kamp-Lintfort, Germany) and 2.5 mL (1:500 ratio of mass to volume) of 6 M hydrochloric acid (> 99%, Alfa Aesar, Kandel, Germany) were added. The vials were closed and kept in a UT 5042 drying oven (Heraeus, Hanau, Germany) at 110 °C for 24 h. The hydrolysate was filtered (0.2 µm PTFE filter), evaporated to dryness under vacuum at 40 °C, reconstituted in 1 mL distilled water and filtered again into small 1.5 mL HPLC vials. The vials were frozen at − 20 °C until LC-IRMS analysis. Glutamine and asparagine are converted to their respective acidic form during this treatment and measured together with glutamic and aspartic acid. Tryptophan and cysteine are lost during acid hydrolysis and the amount of methionine was too low to be measured accurately.

### LC-IRMS analysis of AAs

The analysis of individual AAs was performed on a Dionex Ultimate 3000 HPLC Pump (Thermo Fisher Scientific, Bremen, Germany) coupled to an Isolink^TM^ Interface and Delta V Advantage mass spectrometer (Thermo Fisher Scientific, Bremen, Germany). Separation was achieved for 13 AAs (Fig. S1) with a mixed mode cation exchange column (Primesep A, 2.1 mm ID, 250 mm L, 5 µm particle size) from SIELC, which was in accordance with other studies employing the same separation technique^44, 45, 68^. The exact program is described by a study from Raghaven et al. (2010)^45^ and uses a gradient from mobile phase A (100% water, pH 7) to mobile phase B (0.3 M sulfuric acid, pH 1.5) and column temperature was held at 30 °C. To preserve the HPLC column, which is very sensitive to pH values of over 7, we adjusted the method to start with water at pH 4 as mobile phase A. The flow rate of the mobile phase was set to 260 µLmin^-1^ and oxidation agents (1.5 M orthophosphoric acid and 100 gL^-1^ disodium peroxodisulfate, Merck, Darmstadt, Germany) were pumped at 25 µLmin^-1^ each. This resulted in an oxygen background of approximately 12 V on the first cup of the IRMS, which is the recommended value by the manufacturer to guarantee sufficient oxidation conditions. The experimental units were replicates of pooled dietary samples (n = 3) and biological replicates of stickleback tissues (liver and muscle, n = 5) for each of the four sampling days taken at day 30, 60, 90 and 120 and the small sample amount of obtained stickleback tissue did not allow for within-individual replicate analysis. Each hydrolyzed sample was injected in triplicate into the LC-IRMS system and outliers were determined by Grubbs tests on a confidence level of 0.95 and excluded from further analysis. We calculated mean values and SD from triplicate injections to estimate instrumental precision before referencing our data. Instrumental precision was estimated with an average SD of 0.47‰ for triplicate injections of all AAs, tissues, and sampling days (n = 674). SDs of triplicate injections of all AAs from either dietary samples (0.51‰, n = 156), liver samples (0.50‰, n = 258) and muscle samples (0.41‰, n = 260) where almost equal over the complete sampling period. The robustness of sample preparation and hydrolysis was assessed by conducting replicate analysis (n = 3) of dietary samples, since these were the only samples providing enough material for multiple replicates. The average SD of replicate analysis was 0.36‰ and therefore in the same range as triplicate injections. Twelve in-house amino acid standards (Ala, Asx, Arg, Glx, Gly, His, Lys, Pro, Phe, Ser, Thr and Tyr) were purchased with a purity of > 98% (Alfa Aesar, Kandel, Germany) and measured against seven certified international AA reference materials (L-Alanine, L-Glutamic acid, USGS 64, USGS 66, L-Phenylalanine, L-Proline and L-Valine), purchased from Arndt Schimmelmann, Department of Earth and Atmospheric Sciences at Indiana University (Bloomington, IN, USA), on an Isoprime100 Elemental Analyser (Elementar Analysensysteme GmbH, Langenselbold, Germany). A mix of the in-house standards with a concentration of 100 mgL^-1^ for each amino acid was regularly measured in between sample runs and used to directly assign final isotope values on the VPDB scale. Measurements of pooled AA in-house standards were further monitored to ensure equal system and method performance throughout the prolonged measurement periods and the SD of AA standards never exceeded 0.6‰ (N = 34, 17 and 20, respectively; Table S5). This procedure ensures accurate long-term isotope data and follows the identical treatment procedure^69^. Five primary RM were measured on the LC-IRMS system before the start of a four week-long measurement (10 and 5 µL injection volumes, each in triplicate). The measured δ^13^C values of the five RM were calibrated with the AA in-house standards and were in good agreement with the true literature values except for USGS 41, which showed conversion of glutamic acid to pyroglutamic acid and is a known problem even with the newer USGS 41a material^70^. Chromatograms were individually checked for proper background and peak detection. The automated dynamic background detection algorithm from ISODAT 2.0 software with a block width of 1000^71^ was in many cases able to accurately estimate the background signal, but manual adjustments had to be made, e.g. for closely eluding AAs or interference of matrix components.

### Data analysis

Data analysis was done using Excel from Microsoft Office 365 ProPlus (Microsoft, Redmond, Washington, USA), Origin 2019 version 9.60 (OriginLab, Northampton, Massachusetts, USA) and Matlab R2021a (MathWorks Inc., Natick, Massachusetts, USA) with the PLS_Toolbox suite (Eigenvector Research Inc., Manson, WA). Isotope data are reported as mean δ^13^C_AA_ values on the VPDB scale in ‰ with its corresponding standard deviation (Table S1). Data was tested for normality with Kolmogorov–Smirnov tests on a confidence level of 0.95, which was not violated for any given AA and sample. One-way ANOVA was used on δ^13^C values of each tissue to test for differences of AAs between sampling days (fixed factor). Using sampling days as an independent variable in ANOVA can be problematic because it is not strictly categorial, but each of our sample represents an independent fish individual which couldn’t be sampled multiple times and is therefore not a repeated measure. We further conducted ASCA as a multivariate analysis in a design of experiment approach, with sampling days and tissue types as fixed factors and δ^13^C of each AA as multivariable. ASCA combines the principles of ANOVA and PCA^65^ and allows to investigate differences in the isotope signature between tissues without the influence of the expected isotope shift over time dominating our analysis. δ^13^C data for ASCA was used without preprocessing and the analysis performed with 1000 permutations.

Δδ^13^C values of individual AAs were first calculated individually between each stickleback sample (muscle, liver) and dietary sample and then averaged over all samples on either (1) all days or (2) each sampling day (Table S3). Δδ^13^C were pooled for NEAAs and EAAs to test for differences in trophic fractionation between those groups among all days. Two-sided t-tests on Δδ^13^C values of individual AAs were used to test for differences in trophic fractionation against 0‰ on individual sampling days. The significance level α was set to 0.01 to compensate for the low number of biological replicates (n = 5 for fish tissue) that were analyzed, and each ANOVA analysis was accompanied by Brown-Forsythe tests (α = 0.05) to check for equality of group variances and followed up with Tukey’s post hoc tests to identify significant differences between group values. Reducing the significance level avoids false-positive results for small sample sizes, but it consequently increases false-negative results, and we are therefore only discussing the most significant differences of our data, while smaller differences might be lost.

## Supplementary Information

Supplementary Information.

## Data Availability

The datasets generated during and/or analysed during the current study are available in the Figshare repository: https://doi.org/10.6084/m9.figshare.17014220.v1.
