# Supplementary material for: Insights into amino acid fractionation and incorporation by compound-specific carbon isotope analysis of three-spined sticklebacks
Source: Sci Rep. 2022 Jul 8;12:11690. doi: 10.1038/s41598-022-15704-7 (PMC9270445; doi:10.1038/s41598-022-15704-7)
Supplement: Supplementary file 1 — Supplementary Information. [file 41598_2022_15704_MOESM1_ESM.docx]

**Insights into amino acid fractionation and incorporation by compound-specific carbon isotope analysis of three-spined sticklebacks**

Authors:

Tobias Hesse^1^, Milen Nachev^2,3^, Shaista Khaliq^1^, Maik A. Jochmann*^,1,3^, Frederik Franke^4,6^, Jörn P. Scharsack^4,5^, Joachim Kurtz^4^, Bernd Sures^2,3^, Torsten C. Schmidt^1,3^

Affiliations:

1. Instrumental Analytical Chemistry, University of Duisburg-Essen, Universitätsstr. 5, 45141 Essen, Germany
2. Aquatic Ecology, University of Duisburg-Essen, Universitätsstr. 5, 45141 Essen, Germany
3. Centre for Water and Environmental Research, University of Duisburg-Essen, Universitätsstr. 5, 45141 Essen, Germany
4. Institute for Evolution & Biodiversity, University of Münster, Hüfferstr. 1, 48149 Münster, Germany
5. Present address: Thünen Institute of Fisheries Ecology, Herwigstr. 31, 27572 Bremerhaven, Germany
6. Present address: Bavarian State Institute of Forestry, Hans-Carl-von-Carlowitz-Platz 1, 85354 Freising, Germany

Keywords: amino acids, compound-specific stable isotope analysis, feeding experiment, metabolic pathways, stickleback

Figure S1: Chromatographic separation of 13 AAs from fish muscle. Val was excluded from this study because it coeluted with an unknown species in dietary samples. Met and Cys were not measurable after hydrolysis due to decomposition or low abundance.

Table S1: Average AA δ^13^C values and standard deviations (SD) in ‰ for each sampling day of dietary (n = 3), liver (n = 5) and muscle (n = 5) samples.

| **Tissue** | **Sampling Day** | **Ala** | | **Asp** | | **Glu** | | **Gly** | | **Pro** | | **Ser** | |
| --- | --- | --- | --- | --- | --- | --- | --- | --- | --- | --- | --- | --- | --- |
|  |  | Avg | SD | Avg | SD | Avg | SD | Avg | SD | Avg | SD | Avg | SD |
| Diet | 30 | -11.8 | 0.1 | -11.0 | 0.1 | -11.4 | 0.6 | -6.0 | 0.3 | -12.5 | 0.5 | -1.1 | 0.4 |
|  | 60 | -13.4 | 0.4 | -12.2 | 0.4 | -12.4 | 0.5 | -7.2 | 0.4 | -13.9 | 0.3 | -2.8 | 0.7 |
|  | 90 | -16.0 | 0.4 | -14.4 | 0.5 | -14.1 | 0.3 | -8.8 | 0.1 | -14.8 | 0.7 | -4.5 | 0.4 |
|  | 120 | -14.5 | 0.3 | -13.1 | 0.4 | -12.7 | 0.3 | -7.6 | 0.5 | -13.9 | 0.3 | -3.3 | 0.4 |
| Liver | 30 | -13.1 | 0.9 | -11.8 | 1.1 | -11.9 | 1.0 | -7.1 | 1.0 | -13.7 | 1.5 | -3.8 | 1.1 |
|  | 60 | -15.3 | 0.9 | -12.8 | 0.8 | -13.9 | 0.8 | -7.3 | 0.8 | -14.8 | 0.8 | -4.9 | 1.4 |
|  | 90 | -15.4 | 1.0 | -14.8 | 1.1 | -14.5 | 1.6 | -7.0 | 0.9 | -14.7 | 1.8 | -5.8 | 1.0 |
|  | 120 | -14.8 | 0.9 | -14.2 | 0.9 | -13.3 | 0.7 | -5.9 | 0.7 | -14.0 | 1.1 | -4.4 | 0.7 |
| Muscle | 30 | -12.4 | 1.0 | -11.7 | 1.0 | -11.8 | 0.9 | -7.9 | 1.1 | -13.9 | 0.9 | -2.1 | 1.1 |
|  | 60 | -12.7 | 0.6 | -11.9 | 0.6 | -11.9 | 0.3 | -7.6 | 0.5 | -14.5 | 0.7 | -0.9 | 0.6 |
|  | 90 | -13.5 | 0.3 | -12.7 | 0.5 | -12.4 | 0.7 | -8.7 | 0.5 | -15.1 | 0.8 | -2.5 | 0.6 |
|  | 120 | -13.1 | 0.5 | -12.2 | 0.4 | -12.2 | 0.4 | -7.5 | 0.8 | -14.0 | 1.0 | -2.7 | 0.8 |
| **Tissue** | **Time Point** | **Arg** | | **His** | | **Lys** | | **Phe** | | **Thr** | | **Tyr** | |
|  |  | Avg | SD | Avg | SD | Avg | SD | Avg | SD | Avg | SD | Avg | SD |
| Diet | 30 | -17.2 | 0.3 | -15.4 | 0.6 | -14.5 | 0.4 | -22.1 | 0.1 | -10.8 | 0.3 | -19.2 | 0.7 |
|  | 60 | -18.3 | 0.3 | -16.3 | 0.3 | -15.7 | 0.3 | -23.4 | 0.1 | -12.6 | 0.3 | -20.4 | 0.5 |
|  | 90 | -19.4 | 0.4 | -17.7 | 0.9 | -17.5 | 0.3 | -25.0 | 0.5 | -14.0 | 0.2 | -21.5 | 0.3 |
|  | 120 | -18.5 | 0.6 | -16.4 | 0.7 | -16.6 | 0.3 | -24.3 | 0.1 | -12.5 | 0.1 | -21.5 | 0.0 |
| Liver | 30 | -16.8 | 0.5 | -8.6 | 0.5 | -14.6 | 0.4 | -23.8 | 0.6 | -10.4 | 0.7 | -19.9 | 0.4 |
|  | 60 | -18.1 | 0.4 | -7.8 | 0.9 | -16.2 | 0.4 | -24.6 | 0.7 | -11.7 | 0.7 | -21.4 | 0.4 |
|  | 90 | -19.0 | 1.0 | -9.0 | 1.1 | -16.7 | 0.8 | -25.1 | 0.8 | -12.6 | 1.4 | -22.9 | 1.3 |
|  | 120 | -18.9 | 0.3 | -7.6 | 0.8 | -17.2 | 0.6 | -26.0 | 0.6 | -11.6 | 1.0 | -23.2 | 0.5 |
| Muscle | 30 | -18.2 | 1.0 | -11.6 | 1.1 | -15.3 | 0.9 | -23.8 | 0.8 | -11.4 | 1.0 | -21.6 | 1.2 |
|  | 60 | -18.3 | 0.8 | -11.4 | 0.7 | -15.7 | 0.3 | -24.4 | 0.6 | -11.4 | 0.9 | -21.9 | 0.6 |
|  | 90 | -19.2 | 0.6 | -11.1 | 0.9 | -16.2 | 0.4 | -25.0 | 0.4 | -11.9 | 0.7 | -22.8 | 0.9 |
|  | 120 | -18.4 | 0.2 | -12.0 | 1.1 | -16.3 | 0.3 | -24.6 | 0.2 | -12.8 | 0.5 | -22.2 | 0.5 |

******

Figure S2: Average δ13C values ± SD (error bars, n = 5) are given in ‰ after 30, 60, 90 and 120 days of the experiment for dietary, muscle and liver samples. δ^13^C values in dietary samples are decreasing over the first 90 days of the experiment, which also causes a significant decrease in δ^13^C values of liver samples between day 30 and 90 of the sampling period (one-way ANOVA, p < 0.01, Table S2). The decrease in δ^13^C values of muscle samples was not significant between any sampling date.

Table S2: AAs in the liver show higher variability in their carbon isotope signatures in response to dietary δ^13^C shifts over time. F-values and average differences between δ^13^C values (dependent variable) on each sampling day (independent variable) are given from one-way ANOVA (DF = 3, 8) and Tukey tests for each individual AA and tissue. Δδ^13^C values are given in ‰ with their p-values in brackets and significant differences (p < 0.01) are marked italic bold. All dietary AAs except His have significantly different δ^13^C values between sampling days, which are mostly driven by differences between 30 and 90 days. AA δ^13^C values in the liver are significantly different for Ala, Asx, Arg, Lys, Phe and Tyr and are also mainly driven by differences between 30 and 90, with the addition of Glx between those specific days. The AA δ^13^C values in the muscle are not significantly different between any sampling days.

| **Tissue** | **AA** | **F - value** | | **Δδ^13^C_60-30_** | | **Δδ^13^C_90-30_** | | **Δδ^13^C_90-60_** | | **Δδ^13^C_120-30_** | | **Δδ^13^C_120-60_** | | **Δδ^13^C_120-90_** | |
| --- | --- | --- | --- | --- | --- | --- | --- | --- | --- | --- | --- | --- | --- | --- | --- |
| **Diet** | Ala | ***84.2*** | ***(0.000)*** | ***-1.6*** | ***(0.002)*** | ***-4.2*** | ***(0.000)*** | ***-2.6*** | ***(0.000)*** | ***-2.7*** | ***(0.000)*** | -1.1 | (0.017) | ***1.5*** | ***(0.003)*** |
|  | Asx | ***44.7*** | ***(0.000)*** | -1.2 | (0.015) | ***-3.4*** | ***(0.000)*** | ***-2.2*** | ***(0.000)*** | ***-2.1*** | ***(0.001)*** | -0.9 | (0.082) | 1.3 | (0.011) |
|  | Glx | ***20.5*** | ***(0.000)*** | -1.0 | (0.078) | ***-2.8*** | ***(0.000)*** | ***-1.8*** | ***(0.005)*** | -1.3 | (0.024) | -0.3 | (0.826) | 1.4 | (0.016) |
|  | Gly | ***35.9*** | ***(0.000)*** | -1.2 | (0.011) | ***-2.8*** | ***(0.000)*** | ***-1.6*** | ***(0.002)*** | ***-1.6*** | ***(0.002)*** | -0.4 | (0.462) | 1.2 | (0.010) |
|  | Pro | ***11.9*** | ***(0.003)*** | -1.4 | (0.029) | ***-2.3*** | ***(0.002)*** | -0.9 | (0.170) | -1.4 | (0.030) | 0.0 | (1.000) | 0.9 | (0.165) |
|  | Ser | ***24.3*** | ***(0.000)*** | -1.7 | (0.014) | ***-3.4*** | ***(0.000)*** | -1.7 | (0.012) | ***-2.2*** | ***(0.003)*** | -0.5 | (0.614) | 1.2 | (0.066) |
|  | Tyr | ***19.3*** | ***(0.001)*** | -1.2 | (0.036) | ***-2.3*** | ***(0.001)*** | -1.1 | (0.064) | ***-2.3*** | ***(0.001)*** | -1.1 | (0.055) | 0.0 | (0.999) |
|  | Arg | ***13.8*** | ***(0.002)*** | -1.1 | (0.051) | ***-2.2*** | ***(0.001)*** | -1.1 | (0.049) | -1.3 | (0.025) | -0.2 | (0.956) | 0.9 | (0.100) |
|  | His | 6.4 | (0.016) | -1.0 | (0.348) | -2.4 | (0.011) | -1.4 | (0.123) | -1.0 | (0.301) | -0.1 | (0.999) | 1.3 | (0.145) |
|  | Lys | ***45.9*** | ***(0.000)*** | -1.2 | (0.010) | ***-3.0*** | ***(0.000)*** | ***-1.8*** | ***(0.001)*** | ***-2.2*** | ***(0.000)*** | -1.0 | (0.027) | 0.8 | (0.060) |
|  | Phe | ***68.6*** | ***(0.000)*** | ***-1.3*** | ***(0.001)*** | ***-2.9*** | ***(0.000)*** | ***-1.6*** | ***(0.000)*** | ***-2.2*** | ***(0.000)*** | -0.9 | (0.015) | 0.7 | (0.035) |
|  | Thr | ***92.0*** | ***(0.000)*** | ***-1.8*** | ***(0.000)*** | ***-3.2*** | ***(0.000)*** | ***-1.4*** | ***(0.000)*** | ***-1.8*** | ***(0.000)*** | 0.0 | (1.000) | ***1.4*** | ***(0.000)*** |
| **Liver** | Ala | ***6.8*** | ***(0.004)*** | ***-2.2*** | ***(0.007)*** | ***-2.3*** | ***(0.005)*** | -0.1 | (0.999) | -1.6 | (0.052) | 0.6 | (0.747) | 0.6 | (0.680) |
|  | Asx | ***9.2*** | ***(0.001)*** | -1.0 | (0.453) | ***-3.0*** | ***(0.001)*** | -2.0 | (0.024) | ***-2.4*** | ***(0.009)*** | -1.4 | (0.161) | 0.6 | (0.741) |
|  | Glx | 5.1 | (0.011) | -2.0 | (0.043) | ***-2.6*** | ***(0.009)*** | -0.5 | (0.861) | -1.4 | (0.211) | 0.6 | (0.813) | 1.2 | (0.372) |
|  | Gly | 2.6 | (0.091) | -0.2 | (0.989) | 0.1 | (0.995) | 0.3 | (0.947) | 1.2 | (0.167) | 1.4 | (0.097) | 1.1 | (0.243) |
|  | Pro | 0.7 | (0.577) | -1.0 | (0.650) | -0.9 | (0.713) | 0.1 | (1.000) | -0.2 | (0.993) | 0.8 | (0.801) | 0.7 | (0.853) |
|  | Ser | 3.2 | (0.054) | -1.1 | (0.425) | -2.0 | (0.042) | -0.9 | (0.611) | -0.6 | (0.826) | 0.5 | (0.872) | 1.4 | (0.191) |
|  | Tyr | ***20.8*** | ***(0.000)*** | -1.5 | (0.024) | ***-2.9*** | ***(0.000)*** | -1.4 | (0.035) | ***-3.3*** | ***(0.000)*** | ***-1.8*** | ***(0.007)*** | -0.4 | (0.853) |
|  | Arg | ***13.7*** | ***(0.000)*** | -1.2 | (0.023) | ***-2.1*** | ***(0.000)*** | -0.9 | (0.127) | ***-2.1*** | ***(0.000)*** | -0.8 | (0.159) | 0.1 | (0.999) |
|  | His | 2.8 | (0.077) | 0.8 | (0.530) | -0.4 | (0.856) | -1.2 | (0.189) | 1.0 | (0.341) | 0.2 | (0.994) | 1.4 | (0.095) |
|  | Lys | ***18.4*** | ***(0.000)*** | ***-1.6*** | ***(0.003)*** | ***-2.1*** | ***(0.000)*** | -0.5 | (0.559) | ***-2.6*** | ***(0.000)*** | -1.0 | (0.065) | -0.5 | (0.522) |
|  | Phe | ***8.9*** | ***(0.001)*** | -0.8 | (0.281) | -1.3 | (0.040) | -0.5 | (0.689) | ***-2.2*** | ***(0.001)*** | -1.4 | (0.026) | -0.9 | (0.201) |
|  | Thr | 4.4 | (0.019) | -1.3 | (0.179) | -2.3 | (0.011) | -0.9 | (0.478) | -1.2 | (0.253) | 0.1 | (0.996) | 1.1 | (0.362) |
| **Muscle** | Ala | 2.8 | (0.071) | -0.3 | (0.901) | -1.1 | (0.070) | -0.8 | (0.230) | -0.8 | (0.294) | -0.5 | (0.666) | 0.3 | (0.830) |
|  | Asx | 1.9 | (0.172) | -0.2 | (0.983) | -0.9 | (0.167) | -0.8 | (0.294) | -0.5 | (0.674) | -0.3 | (0.864) | 0.5 | (0.718) |
|  | Glx | 1.0 | (0.440) | -0.2 | (0.976) | -0.6 | (0.431) | -0.4 | (0.670) | -0.4 | (0.711) | -0.3 | (0.910) | 0.2 | (0.962) |
|  | Gly | 2.6 | (0.090) | 0.3 | (0.944) | -0.8 | (0.336) | -1.1 | (0.141) | 0.4 | (0.872) | 0.1 | (0.997) | 1.2 | (0.098) |
|  | Pro | 1.9 | (0.175) | -0.6 | (0.744) | -1.1 | (0.204) | -0.6 | (0.719) | 0.0 | (1.000) | 0.5 | (0.792) | 1.1 | (0.235) |
|  | Ser | 5.0 | (0.013) | 1.2 | (0.139) | -0.4 | (0.851) | -1.6 | (0.031) | -0.6 | (0.611) | -1.8 | (0.013) | -0.2 | (0.971) |
|  | Tyr | 1.9 | (0.170) | -0.3 | (0.946) | -1.2 | (0.145) | -0.9 | (0.340) | -0.6 | (0.685) | -0.3 | (0.940) | 0.6 | (0.658) |
|  | Arg | 2.2 | (0.133) | -0.1 | (0.992) | -1.0 | (0.146) | -0.9 | (0.232) | -0.2 | (0.981) | 0.0 | (1.000) | 0.8 | (0.269) |
|  | His | 0.7 | (0.541) | 0.1 | (0.994) | 0.5 | (0.847) | 0.3 | (0.941) | -0.4 | (0.911) | -0.5 | (0.801) | -0.9 | (0.475) |
|  | Lys | 3.7 | (0.034) | -0.4 | (0.573) | -0.9 | (0.075) | -0.5 | (0.552) | -1.0 | (0.040) | -0.6 | (0.369) | -0.1 | (0.987) |
|  | Phe | 4.0 | (0.027) | -0.5 | (0.461) | -1.2 | (0.017) | -0.7 | (0.267) | -0.7 | (0.200) | -0.2 | (0.935) | 0.5 | (0.568) |
|  | Thr | 3.4 | (0.044) | 0.0 | (1.000) | -0.5 | (0.728) | -0.6 | (0.698) | -1.4 | (0.064) | -1.4 | (0.058) | -0.9 | (0.359) |

Table S3: Essential and non-essential AAs are mostly rooted from dietary sources in liver and muscle tissue, with average trophic fractionation values (Δδ^13^C) below ± 1.2 ‰. Δδ^13^C values were calculated between fish tissue (liver/muscle) and diets for all days (N = 20) and on each sampling day (N = 5). Values are given in ‰ as average Δδ^13^C (SD) and bold italic numbers indicate significant differences from 0‰ (two-sided t-tests, α = 0.01), which were found in liver tissue for Ala, Asx, Glx, Gly, Ser, Tyr and Phe. Differences for those AAs in the liver were between -2.0 to +1.8 ‰ compared to diets and Gly was the only AA in the liver to have positive Δδ13C values. Δδ^13^C values in muscle tissue are significantly different from 0‰ for Ala, Asx, Glx, Ser, Lys and Thr mostly after 90 days and caused by a lack of response in muscle tissue to the dietary isotope shift. An overall exception is His, which shows high Δδ^13^C values in liver (+8.2 ± 1.2‰) and muscle (+ 4.9 ± 1.4‰).

| **AA** | **Δδ^13^C_Liver-Diet_** | | | | | | | | | | **Δδ^13^C_Muscle-Diet_** | | | | | | | | | |
| --- | --- | --- | --- | --- | --- | --- | --- | --- | --- | --- | --- | --- | --- | --- | --- | --- | --- | --- | --- | --- |
|  | **All Days** | | **30 Days** | | **60 Days** | | **90 Days** | | **120 Days** | | **All Days** | | **30 Days** | | **60 Days** | | **90 Days** | | **120 Days** | |
| **Ala** | -0.8 | (1.3) | -1.4 | (0.9) | ***-2.0*** | ***(0.9)*** | 0.5 | (1.0) | -0.3 | (0.9) | ***1.0*** | ***(1.3)*** | -0.6 | (1.0) | 0.7 | (0.6) | ***2.5*** | ***(0.3)*** | ***1.3*** | ***(0.5)*** |
| **Asx** | ***-0.8*** | ***(1.0)*** | -0.9 | (1.1) | -0.6 | (0.8) | -0.5 | (1.1) | -1.1 | (0.9) | 0.5 | (1.1) | -0.8 | (1.0) | 0.3 | (0.6) | ***1.7*** | ***(0.5)*** | 0.8 | (0.4) |
| **Glx** | ***-0.8*** | ***(1.1)*** | -0.5 | (1.0) | -1.5 | (0.8) | -0.3 | (1.6) | -0.6 | (0.7) | 0.6 | (1.0) | -0.4 | (0.9) | 0.5 | (0.3) | ***1.8*** | ***(0.7)*** | 0.5 | (0.4) |
| **Gly** | 0.6 | (1.5) | -1.1 | (1.0) | -0.1 | (0.8) | ***1.8*** | ***(0.9)*** | ***1.7*** | ***(0.7)*** | -0.5 | (1.1) | -1.9 | (1.1) | -0.4 | (0.5) | 0.1 | (0.5) | 0.1 | (0.8) |
| **Pro** | -0.5 | (1.4) | -1.2 | (1.5) | -0.8 | (0.8) | 0.2 | (1.8) | -0.1 | (1.1) | -0.6 | (1.0) | -1.4 | (0.9) | -0.6 | (0.7) | -0.3 | (0.8) | -0.1 | (1.0) |
| **Ser** | ***-1.8*** | ***(1.2)*** | ***-2.8*** | ***(1.1)*** | -2.2 | (1.4) | -1.3 | (1.0) | -1.1 | (0.7) | 0.9 | (1.5) | -1.0 | (1.1) | ***1.9*** | ***(0.6)*** | ***2.0*** | ***(0.6)*** | 0.6 | (0.8) |
| **Tyr** | ***-1.2*** | ***(0.8)*** | ***-0.7*** | ***(0.4)*** | ***-1.0*** | ***(0.4)*** | -1.4 | (1.3) | ***-1.7*** | ***(0.5)*** | ***-1.5*** | ***(1.0)*** | -2.4 | (1.2) | ***-1.5*** | ***(0.6)*** | -1.3 | (0.9) | -0.7 | (0.5) |
| **Arg** | 0.1 | (0.7) | 0.4 | (0.5) | 0.2 | (0.4) | 0.4 | (1.0) | -0.5 | (0.3) | -0.2 | (0.8) | -1.0 | (1.0) | -0.1 | (0.8) | 0.2 | (0.6) | 0.1 | (0.2) |
| **His** | ***8.2*** | ***(1.2)*** | ***6.8*** | ***(0.5)*** | ***8.5*** | ***(0.9)*** | ***8.7*** | ***(1.1)*** | ***8.7*** | ***(0.8)*** | ***4.9*** | ***(1.4)*** | ***3.8*** | ***(1.1)*** | ***4.9*** | ***(0.7)*** | ***6.6*** | ***(0.9)*** | ***4.4*** | ***(1.1)*** |
| **Lys** | -0.1 | (0.8) | -0.1 | (0.4) | -0.5 | (0.4) | 0.8 | (0.8) | -0.5 | (0.6) | 0.2 | (0.9) | -0.8 | (0.9) | -0.1 | (0.3) | ***1.3*** | ***(0.4)*** | 0.3 | (0.3) |
| **Phe** | ***-1.1*** | ***(0.9)*** | ***-1.7*** | ***(0.6)*** | -1.2 | (0.7) | -0.1 | (0.8) | ***-1.7*** | ***(0.6)*** | ***-0.7*** | ***(0.9)*** | ***-1.7*** | ***(0.8)*** | -0.9 | (0.6) | 0.0 | (0.4) | -0.3 | (0.2) |
| **Thr** | ***0.9*** | ***(1.0)*** | 0.4 | (0.7) | 0.8 | (0.7) | 1.3 | (1.4) | 1.0 | (1.0) | 0.6 | (1.3) | -0.6 | (1.0) | 1.2 | (0.9) | ***2.0*** | ***(0.7)*** | -0.2 | (0.5) |

Table S4: Pooled NEAAs in the liver show negative trophic fractionation values around -0.7 ‰ compared to trophic fractionation close to zero for EAAs in the liver and both NEAAs and EAAs in muscle. All AAs were divided and pooled in NEAAs and EAAs prior analysis. Two-way ANOVA was conducted on Δδ^13^C with NEAA/EAAs and tissue (liver, muscle) as factor, followed by Tukey-tests. Significant differences from ANOVA and Tukey-tests (α = 0.01) are marked in bold italic. His was excluded from the analysis because it showed high fractionation of unknown origin. No differences in trophic fractionation were found in general between NEAAs and EAAs (F_1, 435_ = 5.6, p = 0.019), but there was a significant interaction between tissues and NEAA/EAAs on Δδ^13^C values (F_1, 435_ = 9.4, p = 0.002). The interaction is caused by negative Δδ^13^C values (-0.7 ± 1.3 ‰) of NEAA_Liver_ compared to Δδ^13^C values of -0.1 ± 1.1 ‰, 0.0 ± 1.4 ‰ and 0.0 ± 1.1 ‰ in EAA_Liver_, NEAA_Muscle_ and EAA_Muscle_, respectively.

| **Sample Statistics** | **N** | **Avg Δδ^13^C** | **SD** |
| --- | --- | --- | --- |
| Liver | 220 | -0.5 | 1.3 |
| Muscle | 220 | 0.0 | 1.3 |
| NEAA | 280 | -0.3 | 1.4 |
| EAA | 160 | -0.1 | 1.1 |
| NEAA_Liver_ | 140 | -0.7 | 1.3 |
| EAA_Liver_ | 80 | -0.1 | 1.1 |
| NEAA_Muscle_ | 140 | 0.0 | 1.4 |
| EAA_Muscle_ | 80 | 0.0 | 1.1 |
| **two-way ANOVA** | **DF** | **F-value** | **p-value** |
| Tissue | 1 | 10.0 | ***0.002*** |
| NEAA/EAA | 1 | 5.6 | 0.019 |
| Interaction | 1 | 9.4 | ***0.002*** |
| Error | 435 |  |  |
| **Tukey-Test** | **Δδ^13^C** | **SE** | **p-value** |
| Muscle - Liver | 0.5 | 0.1 | ***0.000*** |
| EAA - NEAA | 0.3 | 0.1 | 0.019 |
| EAA_Liver_ - NEAA_Liver_ | 0.7 | 0.2 | ***0.001*** |
| NEAA_Muscle_ - NEAA_Liver_ | 0.8 | 0.2 | ***0.000*** |
| NEAA_Muscle_ - EAA_Liver_ | 0.1 | 0.2 | 0.941 |
| EAA_Muscle_ - NEAA_Liver_ | 0.7 | 0.2 | ***0.001*** |
| EAA_Muscle_ - EAA_Liver_ | 0.0 | 0.2 | 1.000 |
| EAA_Muscle_ - NEAA_Muscle_ | -0.1 | 0.2 | 0.959 |

Table S5: Measurements of RM and AA standards on the LC-IRMS to calibrate and validate sample measurements. Five international RM were measured via LC-IRMS and the measured δ^13^C values (N=6) were calibrated with the AA in-house standards. Differences between the calibrated and true δ^13^C values were negligible except for USGS41, which showed conversion of Glu to pyroglutamic acid. SD of continuous AA standard measurements on the LC-IRMS during the prolonged measurement periods (N = 34, 17 and 20, respectively) were used to assure equal system and method performance.

| **RM** | **True δ^13^C** | | **Measured δ^13^C (N=6)** | | **Calibrated δ^13^C** | | **Δδ^13^C** |  |
| --- | --- | --- | --- | --- | --- | --- | --- | --- |
|  | Avg (‰) | SD (‰) | Avg (‰) | SD (‰) | Avg (‰) | SD (‰) | (‰) |  |
| L-Alanine | -17.93 | 0.02 | +14.82 | 0.25 | -17.39 | 0.50 | 0.54 |  |
| USGS41 | +37.63 | 0.05 | +72.70 | 0.29 | 39.58 | 0.42 | 1.95 |  |
| L-Glycine | -40.81 | 0.04 | -8.49 | 0.19 | -40.73 | 0.39 | 0.08 |  |
| L-Phenylalanine | -11.20 | 0.02 | +22.46 | 0.13 | -10.57 | 0.49 | 0.63 |  |
| L-Proline | -12.47 | 0.01 | +20.98 | 0.16 | -12.31 | 0.48 | 0.16 |  |
|  |  |  |  |  |  |  |  |  |
| **AA Standard** | **Calibrated δ^13^C** | | **LC-IRMS (N=34)** | | **LC-IRMS (N=17)** | | **LC-IRMS (N=20)** | |
|  | Avg (‰) | SD (‰) | Avg (‰) | SD (‰) | Avg (‰) | SD (‰) | Avg (‰) | SD (‰) |
| Asx | -22.54 | 0.05 | 10.7 | 0.2 | 10.9 | 0.1 | 10.9 | 0.2 |
| Glx | -13.97 | 0.04 | 19.2 | 0.3 | 19.5 | 0.2 | 19.6 | 0.3 |
| Ser | -24.74 | 0.03 | 8.0 | 0.3 | 8.0 | 0.2 | 8.3 | 0.2 |
| Thr | -42.48 | 0.03 | 20.5 | 0.6 | 20.0 | 0.4 | 21.8 | 0.2 |
| Gly | -10.76 | 0.04 | -10.2 | 0.3 | -10.0 | 0.4 | -10.0 | 0.2 |
| Ala | -19.41 | 0.04 | 12.8 | 0.4 | 11.8 | 0.6 | 11.5 | 0.5 |
| Pro | -10.38 | 0.03 | 22.9 | 0.5 | 24.5 | 0.6 | 24.6 | 0.5 |
| Val | -12.21 | 0.04 | 19.3 | 0.3 | 19.4 | 0.3 | 20.2 | 0.4 |
| Tyr | -22.86 | 0.03 | 10.1 | 0.3 | 10.2 | 0.2 | 10.3 | 0.3 |
| Lys | -12.86 | 0.03 | 19.8 | 0.3 | 20.0 | 0.2 | 20.2 | 0.6 |
| His | -15.88 | 0.02 | 16.9 | 0.4 | 17.2 | 0.2 | 17.2 | 0.4 |
| Phe | -12.12 | 0.04 | 20.9 | 0.5 | 21.2 | 0.4 | 21.1 | 0.3 |
| Arg | -12.29 | 0.03 | 21.1 | 0.4 | 21.4 | 0.4 | 21.2 | 0.3 |
